# Characteristics of persons with multiple sclerosis covered by public drug insurance in a Quebec Birth Cohort

**DOI:** 10.1016/j.pmedr.2025.103093

**Published:** 2025-04-30

**Authors:** Yasmine Sadou, Miceline Mésidor, Marie-Claude Rousseau

**Affiliations:** a Centre Armand-Frappier Santé Biotechnologie, Institut national de la recherche scientifique (INRS), Laval, Canada; b Faculty of Pharmacy, Université de Montréal, Montréal, Canada; c Department of Social and Preventive Medicine, Université de Montréal, Montréal, Canada; d Carrefour de l'innovation, Centre de recherche du Centre hospitalier de l'Université de Montréal (CRCHUM), Montréal, Canada

**Keywords:** Administrative data, Public drug coverage, Characteristics, Multiple sclerosis, Quebec

## Abstract

**Objective:**

People living with multiple sclerosis use medications for several indications, but little is known about their prescription drug use in Quebec, notably because the public drug insurance covers only part of the population. We compared the characteristics of those with public drug insurance to those privately covered.

**Methods:**

In a cohort of persons born in 1970–1974, we identified those living with multiple sclerosis by applying a validated algorithm to administrative health data. Individuals with public coverage were those who had at least one covered period after their date of diagnosis between January 1, 1997, and December 31, 2014. We used descriptive statistics to compare sociodemographic and healthcare utilization characteristics by type of coverage.

**Results:**

Among the 1363 persons living with multiple sclerosis, 720 (53 %) were covered by the public drug insurance. Individuals with public drug coverage were younger, more likely to be materially and socially deprived, and had a lower median income than those with private insurance, but otherwise had similar sociodemographic characteristics. The proportion of people who had at least one multiple sclerosis-related visit to a general practitioner (39 % versus 45 %) and hospitalization (6 % versus 3 %) differed among those with public compared to private coverage. However, the utilization of other health services, including neurologist consultations, did not differ by type of drug coverage.

**Conclusion:**

People with multiple sclerosis covered by the public and private drug insurance differed in terms of age, income, deprivation, multiple sclerosis-related visits to a general practitioner and hospitalizations, but not neurologist consultations.

## 1 Introduction

The Canadian heath care system aims at providing universal coverage for all physician and hospital services. However, coverage of prescription drugs outside the hospital setting is not universal (Rachlis et al., 2018). Provinces establish and fund their own drug plans, with important variations across the country. Whereas some provinces (e.g., Quebec) provide universal coverage through either a private or a public plan, other provinces (e.g., Alberta) (Ayodele et al., 2022) have a partially covered population due to eligibility criteria (Sutherland and Dinh, 2017).

In Quebec, the public drug plan initially covered social assistance recipients, their dependents and people aged 65 and over. In 1997, this coverage was expanded to include the entire population. Enrollment in the public drug insurance plan is mandatory for persons who do not have access to private insurance, typically through employment, a spouse, or parental coverage. In 2017, about 44 % of Quebec's population was covered by the public drug insurance plan (Sutherland and Dinh, 2017). Private drug insurance is required to cover at the minimum the same list of medications covered by the public insurance (Morgan et al., 2017). Differences in costs related to dispensing fee and direct patient contributions may exist between the two types of coverages (Chamoun et al., 2022). The Régie de l'assurance maladie du Québec (RAMQ) pharmaceutical services database provides information on medication use only for those covered by the public insurance. This has likely hampered the study of prescription drug use among persons aged less than 65 years living with chronic diseases in Quebec.

Multiple sclerosis is defined as a dysfunction of the immune system which attacks the central nervous system, causing lesions (Cotsapas et al., 2018). People living with multiple sclerosis use medications to slow disease progression and to manage comorbidities. There is limited knowledge regarding medication use among individuals with multiple sclerosis in Quebec, primarily due to the above-mentioned partial public coverage of medication costs. A more comprehensive understanding of the similarities and differences between individuals covered by public insurance and those with private insurance is essential for assessing the external validity of studies based on data from the public drug insurance. Furthermore, differences in characteristics between individuals with public versus private insurance may reveal potential selection bias in studies that rely solely on pharmaceutical data from the public health system.

The objective of this brief report is to compare the characteristics of individuals living with multiple sclerosis in Quebec covered by public insurance with those covered by private insurance.

## 2 Methods

We conducted a retrospective cohort study using data from the Quebec Birth Cohort on Immunity and Health, CO·MMUNITY, established in 2017 and including individuals born in the province of Quebec in 1970–1974 (Rousseau et al., 2024). The cohort establishment and subsequent analyses were authorized by the Commission of Access to Information of Quebec (#110267-S) and approved by the research ethics committee of the Institut national de la recherche scientifique (CER-15–377).

### 2.1 Multiple sclerosis definition

We used a definition of multiple sclerosis validated in the Canadian population. The presence of disease was defined as having at least three multiple sclerosis-related hospital or medical claims (International Classification of Diseases Ninth/Ten Revisions codes: 340/G35) (Al-Sakran et al., 2018) during the follow-up period. The diagnosis date was considered as the date of the first hospital/medical claim related to multiple sclerosis or some demyelinating diseases (323, 323.82, 341.0, 341.9, 377.3/G36, G36.0, G36.9, G37, G37.8, H46) (Marrie et al., 2010).

### 2.2 Study population

A total of 1363 persons living with multiple sclerosis were identified in the cohort with diagnosis dates between January 1, 1997, and December 31, 2014. To be considered covered by the public drug insurance, they had to be covered for at least one period after their date of diagnosis. They were otherwise considered as having private drug insurance. The follow-up period began at the date of diagnosis and ended at the earliest of December 31, 2014, or the date of death, if applicable.

### 2.3 Variables

Sociodemographic characteristics were collected, such as sex (male/female), parents' place of birth (Quebec/outside Quebec), age at multiple sclerosis diagnosis (in years), residence area using the second character of the postal code of residence (0 for rural; other values = urban) (Canada Post, 2018), census-based median family income (in Canadian dollars) using the three-digit postal code (Statistics Canada, 2001), and indices of social and material deprivation (in the form of quintiles ranging from very privileged to very deprived). These indices were constructed by taking into account six socio-economic indicators documented in the census and based on the full postal code (six characters) of residence: the proportion of single-parent families, among people aged 15 and over; the proportion of people living alone; the proportion of people who are separated, divorced or widowed; the proportion of people without a high school diploma; the proportion of people in employment; and the average income. The first three indicators constitute the social deprivation index, and the last three, the material deprivation index (Pampalon et al., 2009). Sociodemographic characteristics were defined at the date of diagnosis, except for sex and parents' place of birth.

Multiple sclerosis-related health care utilization characteristics were collected in the year following the date of diagnosis and included length of hospitalization (in days) and number of medical visits to general practitioners, neurologists, and emergency departments.

### 2.4 Analyses

Sociodemographic and health care utilization characteristics of people covered by public and private drug insurance were described using descriptive statistics, including means and standard deviations for continuous variables or medians and interquartile ranges if the normality assumption was not respected, and frequencies for categorical variables. We also calculated the proportions of people who had at least one multiple sclerosis-related health care utilization within the year following the date of diagnosis. To compare differences in characteristics between individuals covered by public and private drug insurance, we used Chi-square tests for categorical variables and Student's *t*-tests for continuous variables. The Wilcoxon-Mann-Whitney test was used when the normality assumption was not met. *P*-values <0.05 were considered as indicating statistically significant differences. Analyses were conducted using R.4.3.1 (R Core Team, Vienna, Austria; 2014).

## 3 Results

Of 1363 persons living with multiple sclerosis, 720 (53 %) had at least one coverage period with the public drug insurance. The remaining 643 (47 %) who were never covered by the public drug insurance over the follow-up were considered as privately insured.

For people with multiple sclerosis who had public drug insurance, the mean total duration of this coverage was 5.9 years (SD = 4.6) over a mean follow-up period of 10.4 years (SD = 4.9). Most people (70 %) had only one period of coverage during their follow-up, with a mean total duration of coverage of 5.5 years (Supplementary data, Fig. A1). Twenty-two percent had two periods of coverage, with a mean duration of 3.3 years per period for a mean total duration of 6.5 years covered. Six percent had three periods of coverage and 2 % had between four and seven periods.

Persons with multiple sclerosis having public drug insurance were predominantly female (74 %) and lived in urban areas (77 %) (Table 1). In addition, 90 % had parents born in Quebec and 3 % was deceased during the follow-up period. These characteristics were generally similar to those of persons with multiple sclerosis having private coverage. However, significant differences were observed for age at diagnosis (32 years among publicly covered persons versus 34 years among privately covered ones), census-based median family income ($54,500 versus $60,900) and material (23 % in the most deprived quintile versus 14 %) and social (22 % versus 13 %) deprivation.

**Table 1:** Comparison of sociodemographic characteristics among persons living with multiple sclerosis by type of coverage for drug insurance, CO·MMUNITY cohort, Quebec, Canada, 1997–2014.

Characteristics	Public coverage (*n* = 720)	Private coverage (*n* = 643)	P-value^a^
	N (%)		
Sex			0.12
Female	535 (74.3)	502 (78.1)	
Male	185 (25.7)	141 (21.9)	
Age (in years)^b^	32.1 (5.0)	34.2 (4.8)	<0.001
Mother's place of birth^c^			0.56
Quebec	632 (90.3)	560 (89.2)	
Outside Quebec	68 (9.7)	68 (10.8)	
Father's place of birth^c^			0.20
Quebec	615 (90.3)	541 (88.0)	
Outside Quebec	66 (9.7)	74 (12.0)	
Residence area^c^, ^d^			0.05
Rural	161 (22.6)	116 (18.1)	
Urban	551 (77.4)	523 (81.9)	
Material deprivation index^c^, ^e^^f^			<0.001
Quintile 1 (most privileged)	84 (14.5)	106 (19.8)	
Quintile 2	99 (17.1)	130 (24.3)	
Quintile 3	127 (22.0)	118 (22.1)	
Quintile 4	134 (23.2)	107 (20.0)	
Quintile 5 (most deprived)	134 (23.2)	73 (13.7)	
Social deprivation index^c^, ^e^,			<0.001
Quintile 1 (most privileged)	119 (20.6)	130 (24.3)	
Quintile 2	110 (19.0)	136 (25.5)	
Quintile 3	99 (17.1)	116 (21.7)	
Quintile 4	122 (21.1)	82 (15.3)	
Quintile 5 (most deprived)	128 (22.1)	70 (13.1)	
Median income ^b,f^ (in Canadian dollars)	54,500 (13,400)	60,900 (15,300)	<0.001
Death	21 (2.9)	14 (2.2)	0.49

Compilation based on data from the © Government of Quebec (2017). The Government of Quebec is not responsible for compilations or interpretation of results.

a Chi-square tests were realised for categorical variables and student's t-tests for continuous variables.

b Values are expressed as mean (standard deviation).

c The sum of counts for mother's place of birth, father's place of birth, residence area, material and social deprivation index is not equal to the total count due to missing values.

d Residence area was defined using the 2nd character of the postal code of residence (0 = rural; other values = urban) (Canada Post, 2018).

e The material and social deprivation indices were created using socio-economic indicators documented in the census (Pampalon et al., 2009).

f Census-based median annual family income (Statistics Canada, 2001).

**Fig. 1:**
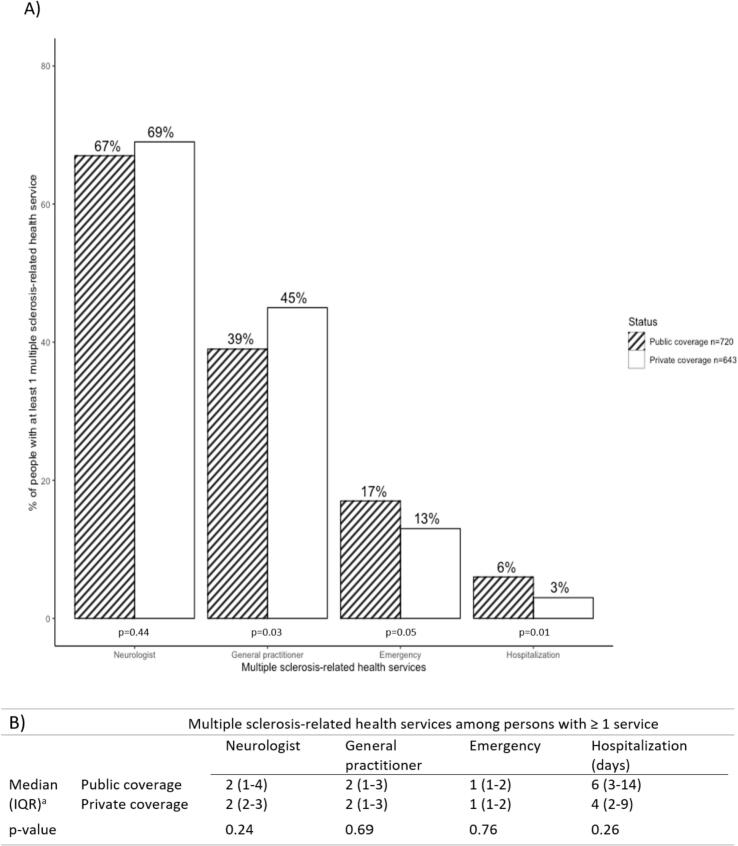
Compilation based on data from the © Government of Quebec (2017). The Government of Quebec is not responsible for compilations or interpretation of results. Multiple sclerosis-related health care utilization in the year after diagnosis among person living with MS, according to the type of drug insurance coverage (public or private), CO·MMUNITY cohort, Quebec, Canada, 1997–2014 (*n* = 1363). Part A corresponds to the proportion of people with at least one multiple sclerosis-related health service among publicly (hatched area) and privately (blank area) covered persons. Part B corresponds to the number of multiple sclerosis-related health services by type of coverage. Chi-square tests were realised for categorical variables (part A) and Wilcoxon-Mann-Whitney tests for continuous variables (part B).

The proportions of people who had at least one multiple sclerosis-related visit to a neurologist (67 % versus 69 %) and emergency department consultations (17 % vs 13 %) were relatively similar between those with public and private coverage (Fig. 1). However, the proportions differed between these groups for multiple sclerosis-related visits to a general practitioner (39 % versus 45 %) and hospitalization (6 % versus 3 %).

Among persons who had at least one multiple sclerosis-related service, the median number of services was almost identical between the two groups.

## 4 Discussion

The aim of our study was to compare the characteristics between persons with public and private drug insurance among people living with multiple sclerosis in a cohort of Quebecers born in 1970–1974. We observed some similarities between the sociodemographic characteristics of these two groups (e.g., sex, residence area and parental birthplace). There were differences in age at diagnosis and some socioeconomic indicators, particularly indices of material and social deprivation and census-based median family income. In terms of multiple sclerosis-related healthcare utilization, neurologist and emergency department consultations were similar, whereas differences were observed for general practitioner consultations and hospitalizations.

In the Quebec population, the proportion of persons covered by the public drug insurance was 28 % among persons aged 25–64 years in 2015–2016 (Sutherland and Dinh, 2017). In comparison, using the closest calendar year from our follow-up, 35 % of our study subjects were publicly covered in 2014. First, the comparison is far from perfect given the wide age range for the population statistic on coverage (25–64 years in the Quebec population versus an average of 43 years in our study). Second, the extent of multiple sclerosis-related disabilities could affect the ability to work and lead to the loss of work-related private insurance in favour of public insurance (Bøe Lunde et al., 2014).

Other studies have been conducted in a population covered by public drug insurance in Quebec, including a cohort study that examined opioid prescriptions in people with chronic non-cancer pain (Racine-Hemmings et al., 2023). Some of the characteristics of their study population appeared to be similar to those of our subjects with public drug insurance, such as a higher proportion of individuals belonging to the highest quintiles of material and social deprivation, which is in line with expectations. Although our results are consistent with those of this study, their population was older than ours (70 % of the population aged ≥65 years).

In terms of health services utilization, predictive factors of multiple sclerosis-specific hospitalizations include sex, age and income (Al-Sakran et al., 2020). The observed differences in our study in multiple sclerosis-related health services use may be attributed to several factors and could also reflect the differential health status of these individuals. However, it is not possible to ascertain whether these differences are a cause or a consequence of public coverage, as the available data is insufficient to make this assessment.

The data used in this study dates to 1997–2014 and newer therapies for multiple sclerosis have since entered the market. Nonetheless, many therapies like interferon-beta and glatiramer acetate remain widely used and reimbursed (Ministère de la Santé et des Services sociaux du Québec, 2025). Reimbursement practices in Canada change slowly and vary by province (Knox et al., 2020; Rawson and Adams, 2017). Thus, the core dynamics of drug access and reimbursement remain applicable to the current context. Further, eligibility criteria for public or private drug insurance coverage have not changed.

Some limitations should be mentioned. The CO·MMUNITY cohort from which the study population was derived is a birth cohort composed of individuals born between 1970 and 1974. The study population is therefore not representative of all persons living with multiple sclerosis in the Quebec population. Another limitation was the use of healthcare services which was restricted to multiple sclerosis-related events and therefore, may not be representative of the overall use of healthcare services of people living with this disease.

This study had several strengths. It is the first study to describe public drug insurance coverage in Quebec among persons living with multiple sclerosis. Considering that drug coverage in the province is universal, i.e., either public or private, our study is also the first to compare the characteristics of these two groups in Quebec among persons living with multiple sclerosis. Additionally, the reliability of the data used is based on the meticulous process of establishing the cohort from which our population was drawn (Rousseau et al., 2024) and the validation in the Canadian population of the algorithm used to identify persons living with multiple sclerosis (Al-Sakran et al., 2018).

In conclusion, this study enabled us to describe the characteristics of people with multiple sclerosis covered by public drug insurance and compare them with those of people privately covered. Our findings can contribute to the evaluation of the external validity of studies examining medication use among individuals under the age of 65 and therefore, providing valuable guidance for researchers and decision makers using and interpreting this type of data.

## Data statement

According to ethical approval and data access authorization, data cannot be shared.

## CRediT authorship contribution statement

**Yasmine Sadou:** Writing – review & editing, Writing – original draft, Visualization, Software, Methodology, Formal analysis, Data curation. **Miceline Mésidor:** Writing – review & editing, Validation, Supervision, Resources, Project administration, Methodology, Conceptualization. **Marie-Claude Rousseau:** Writing – review & editing, Validation, Supervision, Resources, Project administration, Methodology, Conceptualization.

## Funding sources

The establishment of the CO·MMUNITY cohort was supported by the Canada Foundation for Innovation & the Quebec Ministry of Education, Leisure and Sports [grant number 12532], the 10.13039/501100000024Canadian Institutes of Health Research [grant numbers MOP-97777, MCH-97593, MOP-142705, PJT-159791], Fonds de recherche du Québec-Santé (FRQS) [grant number 16227], the 10.13039/501100000261Multiple Sclerosis Society of Canada [grant number 2435], and the 10.13039/501100000015Canadian Cancer Society [grant number 703309]. Y.S. was supported by 10.13039/501100004489Mitacs [grant number IT39277].

## Declaration of competing interest

The authors declare that they have no known competing financial interests or personal relationships that could have appeared to influence the work reported in this paper.

## Appendix A Supplementary data

Supplementary material Image 1
