# Supplementary material for: Characteristics of persons with multiple sclerosis covered by public drug insurance in a Quebec Birth Cohort
Source: Prev Med Rep. 2025 Apr 30;54:103093. doi: 10.1016/j.pmedr.2025.103093 (PMC12135425; doi:10.1016/j.pmedr.2025.103093)
Supplement: Supplementary file 1 — Supplementary material [file mmc1.docx]

**Supplementary data**


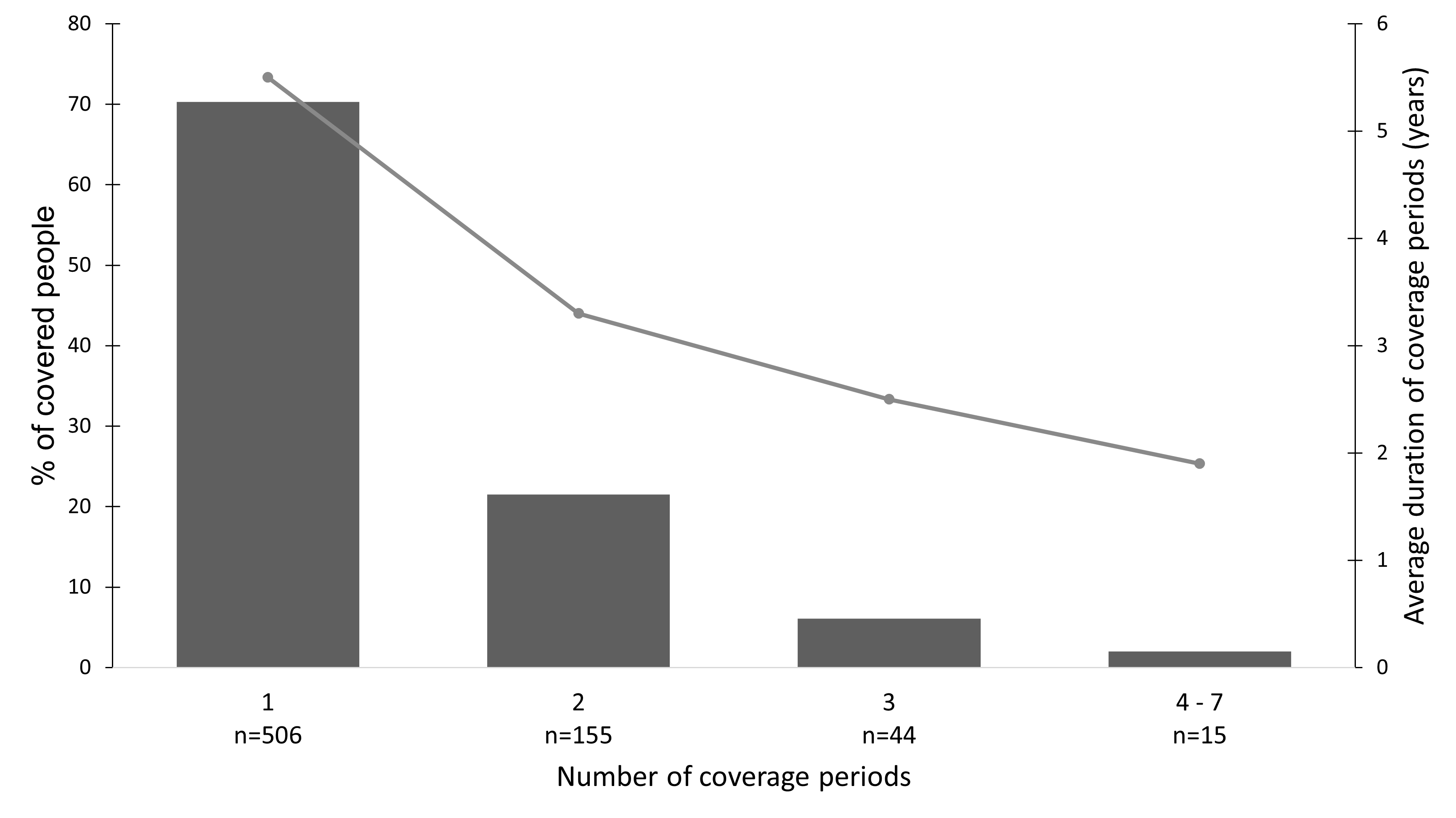


Compilation based on data from the © Government of Quebec (2017). The Government of Quebec is not responsible for compilations or interpretation of results.

Figure A1: Proportion of persons with multiple sclerosis covered by public drug insurance and average coverage duration by number of coverage periods, CO·MMUNITY cohort, Quebec, Canada, 1997-2014 (n=720)

Figure A1 Legend:

The bar chart corresponds to the percentage of covered people (axis on the left). The curve corresponds to the average duration of coverage periods (axis on the right). For example, 70% of the population had one coverage period and an average duration of 5.5 years per coverage period.
